# Persesqui-nitrate of Iron in Chronic Diarrhœa

**Published:** 1876-07

**Authors:** William Kerr

**Affiliations:** Galt


### Selections.

### PERSESQUI-NITRATE OF IRON IN CHRONIC DIARRHŒA.

By WILLIAM KERR, M.D., Galt.

In chronic diarrhœa, from want of tone, I know of no remedy equal to the persesqui-nitrate of iron. Dr. Graves, of Dublin, in his Clinical Medicine, 1842, says "you will be consulted by females of a delicate and weakly habit, who frequently exhibit symptoms of nervous derangement, such as palpitation, sleeplessness and headache, who are easily excited or alarmed, and have a tendency to emaciation and paleness, and have little or no appetite. Combined with these spmptoms you find that they have been laboring under diarrhœa for weeks, or even months, and that this, with other causes of debility, has rendered their condition extremely uncomfortable. You will also be informed by the patient that she has tried many remedies without benefit, and that she is extremely anxious to have something done to give relief, and hence it is a matter of importance to be acquainted with any remedy which may be likely to prove serviceable. This form of diarrhœa is of an unmanageable nature, and very seldom amenable to ordinary modes of management. The common astringent remedies totally fail, chalk-mixture, rhatany-root and catechu are useless; and in such cases it has been observed that opium is generally injurious. If you prescribe opium it commonly checks the the disease for a time, but this temporary relief is accompanied by debility, malaise, restlessness, and many uneasy symptoms, and the diarrhœa soon returns, and is as bad as ever. The medicine which I have found most effectual in such cases is the persesqui-nitrate of iron." (Dr. Graves attributes the introduction of this as a medicine to Sir R. Christison, but in his next edition corrects the error.) "With it I have succeeded in curing many cases which had been exceedingly obstinate, and of very considerable duration; the disease having in one instance resisted all the efforts of medical skill for seven months, and in the other for two years. Seven or eight drops of the liquor ferri persesqui-nitratis, (equal to half a teaspoonful of that of the formula in this paper,) gradually increased to twelve or fifteen, was the quantity prescribed in both cases. In the course of four days a slight diminution of the diarrhœa was perceptible; in a fortnight the patients felt much better, and in a month or five weeks it had disappeared altogether. This took place without being followed by any bad effects. There was no swelling of the stomach, no tympanites, no tormina, no restlessness or nervous derangement, the patients recovered their health and strength, and the cure was at once permanent and safe."

Dr. Neligan, in his work on "Medicines," 1847, states that "the persesqui-nitrate of iron is an admirable astringent, possessing also tonic properties. It will be found particularly useful in chronic cases of mucous diarrhœa, when there is much emaciation and loss of appetite. In such I have derived much benefit from its employment, after many other remedies had failed." Dr. Dunglison, on New Remedies, 1846, says: "Kopp administered it with the greatest success in many cases of chronic diarrhœa that had resisted every approved remedy." Dr. Twining (Diseases of India) and others also testify in its favor.

An elderly gentleman holding an important office in the University of Glasgow, having consulted several of the most eminent medical men in that city, and also in Edinburgh, without success, for diarrhœa of long continuance, which was manifestly reducing his strength, proceeded to London to obtain the advice of the leaders of the profession there. The opinion given was that most approved remedies having failed, the disease would slowly but surely prove fatal. On his return to Glasgow he applied to Dr. Laurie, afterwards Professor of Surgery in the University, who gave him persesqui-nitrate of iron. Rapid improvement followed, diarrhœa ceased, and he lived for years afterwards into old age.

A lady in this country, who for sixteen years had suffered from relaxed bowels, was affected with more than ordinary severity and frequency in the summer of 1848. At this time I was consulted, and advised the persesqui-nitrate of iron. After a few doses the complaint ceased, and did not return, the medicine being continued for some time to establish a cure. Various remedies had previously been tried by the advice of medical men both in Canada and Britain, but without benefit.

The persesqui-nitrate of iron is also valuable in ague, along with quinine, while there are paroxysms, and without, in that stage known as dumb ague. (See *Edinburgh Medical Journal*, September, 1851.)

Its strongly astringent taste, unaccompanied by causticity, first led me to make trials of the persesqui-nitrate of iron, and possibly to these qualities it owes much of its efficacy, but the constituents of the acid may likewise have a share; nitrogen may play an important part, as we know it does in diet, and in an acid so readily decomposed the oxygen and nitrogen may be assimilated with and strengthen the living tissue. We are apt to speak of salts of iron as if all depended on the base, yet a moment's reflection tells, that in salts of potash or soda the acid in combination greatly modifies the qualities of the salt. Looking over the foregoing reports, it is impossible to believe that the various eminent medical men, who had given all the most approved remedies for chronic diarrhœa, had neglected the muriate, sulphate or citrate of iron, but they failed to cure till the persesqui-nitrate was used.

I have long prepared the medicine according to the following formula: Mix one ounce and a half of nitric acid with seven and a half of water, both of these quantities by measure; put into this half an ounce of iron wire (No. 17 is a convenient thickness) broken into pieces and arranged so as to reach every portion of the fluid. At the end of ten or twelve hours pour off the solution from the small remainder of wire, and add as much water as will bring the whole to thirty ounces. In my first formula* the acid and iron are respectively three ounces and one,

* Edinburgh *Medical and Surgical Journal*, January, 1832. See also *Glasgow Medical Journal*, 1832, Edinburgh *Medical Journal*, May, 1843, and September, 1851.

and the total, when completed, thirty, a quantity which has been approximately adopted by the United States Dispensary and the British Pharmacopœia, but is liable to the objection that the solution becomes much sooner turbid than in the more dilute solution. The liquid ought to be transparent, and of a beautiful dark red color. In cold weather the process must be carried on in doors, otherwise the solution will be that of proto-nitrate, but in warm weather this is not necessary. The noxious fumes of nitrous acid can be altogether avoided by placing a receiver over the vessel containing the acid and iron, having its lower edge immersed in an inch or so of water. In making the chloride of gold for photographers, the fumes, which have brought fatal pulmonary disease on some individuals, may be similarly condensed. In preparing the persesqui-nitrate the most important object is as little as possible to exceed the temperature necessary to peroxidize the iron. If the acid is less diluted, the weather very warm, or the quantity prepared greater, it is possible that the heat of the liquid may rise so high that the solution will be turbid from the first; on the other hand, if the heat has been kept moderate, the solution may remain transparent the greater part of a year. The proper temperature is most easily preserved by Prof. Proctor's plan.—*Canada Lancet.*

